# An explanation and analysis of how world religions formulate their ethical decisions on withdrawing treatment and determining death

**DOI:** 10.1186/s13010-015-0025-x

**Published:** 2015-03-11

**Authors:** Susan M Setta, Sam D Shemie

**Affiliations:** Department of Philosophy and Religion, Northeastern University, 360 Huntington Ave., Boston, 02115 MA USA; Division of Pediatric Critical Care, and Medical Director, Extracorporeal Life Support Program at Montreal Children’s Hospital, McGill University Health Centre, 2300 Rue Tupper, Montréal, QC H3H 1P3 Canada

**Keywords:** Determination of death, Defining death, Deceased organ and tissue donation, Withdrawing treatment, World religions, Ethics, Judaism, Christianity, Islam, Hinduism, Buddhism

## Abstract

**Introduction:**

This paper explores definitions of death from the perspectives of several world and indigenous religions, with practical application for health care providers in relation to end of life decisions and organ and tissue donation after death. It provides background material on several traditions and explains how different religions derive their conclusions for end of life decisions from the ethical guidelines they proffer.

**Methods:**

Research took several forms beginning with a review of books and articles written by ethicists and observers of Bön, Buddhism, Christianity, Hinduism, Indigenous Traditions, Islam, Judaism, Shinto and Taoism. It then examined sources to which these authors referred in footnotes and bibliographies. In addition, material was gathered through searches of data bases in religious studies, general humanities, social sciences and medicine along with web-based key word searches for current policies in various traditions.

**Results:**

Religious traditions provide their adherents with explanations for the meaning and purpose of life and include ethical analysis for the situations in which their followers find themselves. This paper aims to increase cultural competency in practitioners by demonstrating the reasoning process religions use to determine what they believe to be the correct decision in the face of death.

**Conclusion:**

Patterns emerge in the comparative study of religious perspectives on death. Western traditions show their rootedness in Judaism in their understanding of the human individual as a finite, singular creation. Although the many branches of Western religions do not agree on precisely how to determine death, they are all able to locate a moment of death in the body. In Eastern traditions personhood is not defined in physical terms. From prescribing the location of death, to resisting medical intervention and definitions of death, Eastern religions, in their many forms, incorporate the beliefs and practices that preceded them. Adding to the complexity for these traditions is the idea that death is a process that continues after the body has met most empirical criteria for determining death. For Hinduism and Buddhism, the cessation of heart, brain and lung function is the beginning of the process of dying—not the end.

## Introduction

This paper explores definitions of death from the perspectives of several of the world’s religions. ^a^Unlike modern medicine, religions often define death with reference to unobservable criteria. Roman Catholicism, for example, believes that death happens when the soul leaves the body; Buddhism sees death occurring at a point after the invisible, subtle consciousness leaves the physical body. Because these events are not visible, religions turn to empirical evidence to determine that death has occurred. This paper seeks to explore how religious perspectives define death and determine that it has occurred with application to end of life care and donation of organs and tissues after death. This paper develops the basic features of religious traditions and the ways in which their ethical analysis takes place. It aims to increase cultural competency in practitioners by demonstrating the reasoning process religions use to determine the correct decision in the face of death.

Research for this explication and analysis took several forms beginning with a review of books and articles written by key ethicists in Judaism, and various forms of Christianity, Hinduism, and Buddhism. It then examined sources to which these authors referred in footnotes and bibliographies. In addition, material was gathered through searches of data bases in religious studies, general humanities, social sciences and medicine along with web-based key word searches for current policies in various traditions.

In his personal statement contained within the President’s Council on Bioethics white paper on determinations of death, Edmund Pelligrino noted:

The so-called “definitions” of death fall into two categories: the philosophical and the empirical. The first seek a conceptual understanding of the essential differences between life and death. The second seek to determine the clinical signs, tests, or criteria which separate life and death most accurately [1].

The debate about the specifics of empirical definitions that began in earnest in the mid 1960’s continues today. George Khushf has noted that “in most of the current biomedical literature, the purely biological character of any death concept is simply asserted as a premise, and subsequent reflection attempts to clarify the nature of such a biological concept” ([2], p.339). These biologically based definitions have developed for several reasons. New life sustaining technologies, including artificial heart-lung machines, the ability to transplant life sustaining organs and compliance with the dead donor rule for the purposes of post mortem transplantation, require precision in determining a specific moment when death can be pronounced. Because of the need for biological clarity in understanding the point of death, there are a wide range of articles offering different opinions on how to make appropriate empirical assessments. While there are well published religious perspectives on organ/tissue donation after death, there is a relative paucity of literature regarding religious perspectives on death determination itself.

The diverse and complex religious perspectives of the world’s religious traditions on this issue has not yet been explored in the kind of detail one sees in the scientifically focused literature. Scholars have provided solid scholarship on a range of religious traditions understanding of determining death. For example, individuals such as Damien Keown [3] and Karma Leske Tsomo [4] discuss Buddhist perspectives, Omar Sultan Haque [5] provides material on Islamic ethics and end of life issues, Yitzchok Breitowitz [6] gives an overview of contemporary Jewish perspectives, and Aaron Mackler provides a comparative analysis of Jewish and Roman Catholic Bioethics [7]. Several anthologies, most notably Lucy Bregman’s three volume Religion, Death and Dying includes individual articles on a number of religious traditions. Chaplains Sue Wintz and George Handzbo have produced a Handbook for healthcare professionals containing useful information from a wide range of traditions on a variety of bio-medical ethical issues including determining death and withdrawing support [8]. There is not, however, one source that provides an overview of the ethical decision making that occurs as various religions think about death and determine that it has occurred. This article fills that void by providing a starting point for the analysis of end-of-life determinations. It uses the perspective and methodology of comparative religion to provide an overview of the basic features of individual traditions, to discuss how they view life and death and to explore how they make moral decisions in the face of death.

When confronting issues at the edges of life, religious perspectives can become especially influential because they explain the nature of the human individual, the goal of life, the reasons for death and for most, what happens after the death of the body. Because religions provide a way of interpreting the world, individuals living in the midst of a particular tradition can continue to be influenced by it even if they have stopped believing or practicing.

The category of brain death that predominates in Western allopathic medical systems relies on a definition of death that emerges from a concept of the human being that is heavily influenced by ancient Greek thought and Descartes’ concept, *cogito ergo sum*; both place greater value on the mind and brain than on other bodily organs. Traditions that do not share these influences construe their understandings of death from sources within their cultures and faiths and can reject determinations of death that center on the brain. In the United States, two states, New York and New Jersey, have enacted legal protections for individuals holding religious views that differ from the standard definitions of death.

Until the middle of the twentieth century, death was something observed. Breathing stopped and so did the beating of the heart. Prior to the introduction of mechanical ventilators in the mid-20th century and the evolution of resuscitative measures, a non-brain or circulation formulation was used to determine death. The concept of brain death was influenced by two major advances in health care in the 1960’s: the development of intensive care units with artificial airways and mechanical ventilators that treated irreversible apnea, thus interrupting the natural evolution from brain failure to cardiac death. The concept of brain death also addressed ethical concerns associated with organ donation that arose from the then-new discipline of transplant surgery. The clinical appearance of brain death was first described by French researchers Mollaret and Goulan who coined the term *coma dépassé* in their seminal 1959 work [9] and described “a state beyond coma”, in which loss of consciousness, brain stem reflexes, and spontaneous respiration was associated with absent encephalographic activity.

Mechanical respirators complicated the situation since they could breathe for a patient. The situation of the artificially maintained patient developed. By the latter half of the 20th century a view of death that did not look to breathing or a beating heart prevailed in most American and European hospitals. This view has its root in the 1968 Harvard Ad Hoc Committee on Brain Death, a committee that was formed to address a number of issues including finding a definition of death that would permit the removal of organs before they deteriorated from lack of circulation [10]. Though this definition is not without opponents from various medical, moral, cultural and religious perspectives, it now dominates European and North American medicine.

### Families of religions and definitions of death

The families of religions can be classified in three basic ways: The Western or Abrahamic traditions of Judaism, Christianity, and Islam; Eastern Traditions which include Hinduism, Jainism, and Buddhism, and indigenous faith systems, also called aboriginal, primal or archaic religions (from the Greek *arché*, original). Within these families of religions there are shared ideas about the nature of the human individual and the world; there can be differences, of course, but, basic features define and unite them.

### Western traditions

Judaism, Christianity and Islam trace their roots to a single ancestor, Abraham, and are united by the way in which they construe human life. All three believe the individual to be a unique creation of God whose life begins at a specific point in time; all understand the human as remaining him or herself throughout life and into the afterlife. All three determine death by looking at the cessation of bodily organs, such as the heart, lungs or brain. ^b^To interpret contemporary moral issues, Western traditions rely heavily on their Scriptures and on authoritative interpretations of their sacred texts.

### Judaism

The three largest movements within contemporary Judaism are Orthodox, Conservative, and Reform. All three agree on key theological precepts such as monotheism, reverence for Scripture, and the celebrations of Judaic traditions such as Yom Kippur and Passover. When they differ, the issues center on practice, requirements for ordination and conversion, and interpreting contemporary moral issues. To determine whether an action is compliant with Jewish law, *halacha*, Judaism will turn to Biblical sources and key interpretations. These include the older writings *Mishnah*, and *Talmud*, and contemporary rabbinic rulings called Responsa. These are used in much the same way contemporary secular law turns to legal precedent to analyze current cases. Orthodoxy believes that obedience to Jewish law is mandatory because it comes from God and is the most traditional in their understanding of Biblical mandates. When dealing with complex issues, Orthodox Jews will turn to their Rabbi to interpret and understand the course of action that is in keeping with the Law.

Conservative Jews also believe that Jewish law is binding, but view the law as developing through both God and human interpreters in a process that continues in the present ([11], p.278). Thus, like their Orthodox counterparts, Conservative Jews will use Scripture and tradition and defer to the ruling of their Rabbi, but their rulings are often more flexible than those of Orthodox rabbis ([12], p.3).

Reform Judaism is the most liberal of the three, it champions individual autonomy and believes that Judaism must adapt to the contemporary situation. Though a Reform Jew may consult a Rabbi, the Rabbi’s view is just one factor in determining the morality of a situation. Reform Jews will use extra-*halachic* materials so that Jewish law is just one of several sources that may be used [12]. The decision of the individual and family in determining the morality of an action is allowed. On the issue at hand, determination of death, there are wide variations across all three groups and within them as well.

As there is no central authority in any of these movements, responses to complex moral issues will vary because of cultural considerations such as nationality or generation. Nonetheless, all three groups would agree that all of life is a gift from God and should be protected and maintained, that humans received the gift of life through the breath of God as recorded in both Genesis and Leviticus where God breathes into Adam in order to enliven him. In addition, taking care of the body is an obligation, since the body belongs to God.

Authoritative ancient sources did not face the situation of a patient whose breath stopped but whose heart continued beating for more than a few seconds or minutes at most. So while cessation of breath and the resulting cessation of a heartbeat was the traditional test for death, the ventilator raises new issues. Spontaneous respiration has ceased because of brain death, but the heart continues to beat. The beating heart in the brain dead patient is the focus of considerable contemporary discussion.

Reform and Conservative Judaism overwhelmingly accept neurological criteria and have, according to Elliott Dorff, since shortly after the Harvard Criteria appeared ([11], p.259). But within Orthodox Judaism, there are two competing opinions on the appropriate way to determine that an individual has died. One side accepts neurological criteria as outlined by the Harvard Committee; the other does not, insisting instead that cessation of heartbeat is the only valid criterion [6].

Those within Orthodoxy who accept neurological criteria in determining death draw on a variety of commentaries to support their view. First, they consult an eleventh century ruling of revered interpreter, Rashi who described [13] the appropriate response to a person trapped in a collapsed building on the Sabbath. Rashi advises uncovering the patient’s nose. If there is breath, attempts to free the person from the rubble can continue because the Sabbath may be violated to save a life; if there is no breath, the work should cease. Nineteenth century authority and rabbi, Moshe Sofer, (1762–1839) and twentieth century rabbi, Moshe Feinstein ^c^ understand this to mean that ‘that halachic death depends on cessation of respiration” [14]. This is then combined with a twelfth century text, *The Babylonian Talmud* written by Maimonides [15] that discusses whether decapitated animals that move are alive or dead. The text’s author writes, “even if they move convulsively like the tail of a newt that twitches spasmodically [after being cut off],” they are dead [16]. This pro-neurological group likens the beating heart to spasmodic movement in animals and considers it extraneous movement, not determinant of life. Because there would be no respiration without mechanical support, Feinstein determined that a patient with no spontaneous respiration should be considered dead [17].

In 1987, The Israeli Chief Rabbinical Council drew on these and other interpretations to affirm the use of neurological criteria and noted five prerequisites for establishing the state of brain death:

A. Clear knowledge of the cause of the injury.
B. Absolute cessation of spontaneous breathing.
C. Detailed clinical proof of injury to the brain.
D. Objective proof of destruction of the brain stem by objective scientific tests, such as the electrical brainstem testing.
E. Proof that the absolute cessation of respiration and inactivity of the brain stem continue for at least twelve hours despite full, customary intensive care [18].

The opposition to this ruling relies on some of the same sources to substantiate its own view [19]. Rabbi J. David Bleich, author of books, articles and *Responsa* on this issue^d^, begins his opposition by stating that it is inappropriate to use scientific or medical criteria to make any determination of death, these decisions, he believes are the purview of moral and religious domains [20]. “Medicine,” he says, “does not define death; it defines physiological states” [21]. He pronounces death to be the cessation of all cardiac and respiratory activity and all movement. To make these rulings he draws on the same commentaries the opposition uses, but points to a section they do not use [22] and comes to a different conclusion. “But as long as he lies like an inanimate stone and has no pulse, if afterward breathing ceases, we have only the words of our holy Torah [to rely on and determine] that he is dead [16]. Bleich considers the rhythmic and continuing beating of the heart to be movement that is not comparable to the spasms occurring after decapitation.

The opposition between these two views has endured for more than fifty years; each side produces volumes of materials, *Responsa* and arguments to bolster its view. It remains contentious. In January of 2011, The Rabbinic Council of American, a union of Modern Orthodox Rabbis declared that it would not lend its support to either position but encouraged its members to make their own decisions on which definitions fulfill the requirements of Jewish law. Around the same time, one rabbinate in the United Kingdom reversed earlier rulings and rejected brain death criteria [21].

### Withdrawal of mechanical support: Judaism

Withdrawing mechanical support at any point is at least as controversial in the orthodox community as the criteria used to define death. Even those who favor using neurological criteria find it difficult to discontinue mechanical support, especially the ventilator, even though the patient has met the criteria for brain death. They cite Joseph Karo’s 16th century commentary [22],

Thus it is forbidden to cause the dying person to die more quickly. For example if one is a *goses* (moribund person) for a long time and is unable to expire, it is forbidden to remove the pillows or mattress from underneath him, . . . *However, if there is something causing a hindrance to the soul’s departure, such as if there is a noise near the house such as a woodchopper, or if there is salt on his tongue, or these are delaying the departure of the soul, it is permitted to remove them.-- all medicine is obligatory but if there is an impediment to dying it may be removed*.^e^ (*Italics* added)

The argument is paradoxical, on the one hand, the patient has been determined dead by brain criteria but on the other, the presence of artificially applied respiration clearly trumps brain death. Feinstein, who accepts neurological criteria, sends a mixed signal when he specifically states that respirator should not be turned off even if the person meets the criteria for brain death. He notes that if a clinically dead patient is on a respirator, “it is forbidden to interrupt the respirator.” He suggests checking for signs of spontaneous respiration while machinery is being serviced and restarting the respirator if there is any sign that breathing might occur [23]. Similarly, Rabbi Eliezer Yehudah Waldenberg notes that, “If it is not clear whether the respirator is keeping the patient alive or only ventilating a corpse, the respirator must be maintained” [24]. Turning it off to determine whether there is breathing is not permitted because it may cause the patient’s death “as the movement of a flickering candle causes the flame to be extinguished.” Waldenberg advocates using respirators on timers that automatically turn off and then must be reset manually. Observing the patient at this time will indicate whether there is spontaneous breathing and will prevent the need for medical personel to disconnect the equipment and possibly engage in ‘an act of commission’ that he cannot endorse [25].

There are occasional voices in the orthodox rabbinic community who believe that the respirator and other interventions may be discontinued. Rabbi David HaLevi sees the maintainance of mechanical support, such as a respirator in a dying patient as an impediment which may be removed as the ‘salt on the tongue,’ [26]. Most in the Orthodox community, however, see these interventions as medical treatment and argue that they are obligatory and must be continued despite the fact that death has been determined using neurological criteria.

Most of orthodoxy agrees, however, that there is a point at which treatment may be suspended, but they disagree on when that point is. Bleich has a very restrictive view and states that medical treatment, though usually obligatory, is not required if it is clear that a patient is moribund (*goses*) and will die within 72 hours ([27], p.141-142). He does not believe treatment should stop under other circumstances regardless of the reason. He notes that even the best intention does not matter; the act of discontinuing treatment for anyone other than a *gose*s is still homicide ([28], p.260). Moshe Feinstein, in basic agreement with Bleich on this issue, extends the period of time during which it is appropriate to withdraw treament to a few weeks. Feinstein is concerned, however, that removing a ventilator might cause a patient’s death, so he urges caution, and recommends reconnecting the ventilator if the patient appears to be alive, so there will be no chance of contributing to his death “. . . for even the slightest period of temporary life” is valuable ([29], p.839).

The Conservative tradition shares the perspective that medical care is obligatory up to a point, but extends the period of time in which treatment may be withheld or withdrawn—up to a year or more. Permission to withhold or withdraw medicines is applied more broadly.^g^ The Conservative Movement’s Council on Jewish Law and Standards has adopted new guidelines for stopping treatment. Rabbi Elliott N. Dorff has successfully introduced the category of *terefah*, which applies as soon as someone receives a diagnosis of terminal, incurable illness. “Permission to withhold or withdraw medications and machines” applies as soon as someone receives their diagnosis [30].

While in Orthodox and Conservative Judaism, the debate centers on when treatment may be terminated, Reform Judaism focuses on the therapeutic effectiveness of treatment. “Ineffective therapy ceases to be medicine” and is not required. The decisions in this branch of Judaism are left to the patient and family in consultation with their pysician ([29], p.841).

### Christianity

Christianity developed from the first century Jewish reform movement centered on Jesus. From these roots Christianity inherits respect for scripture and authoritative interpretation and brings both to the examination of contemporary moral issues. As it moved throughout the Roman Empire, Christianity borrowed key ideas from Greek philosophy, especially Plato and Aristotle. Two aspects of Greek influence are important here: the Platonic view that the human is comprised of two separate components, body and soul, and the idea that the mind of the individual is superior to the body.

A schism that began in the early eleventh century separated Christianity into two forms, now called Western Christianity and Eastern Orthodoxy. Western Christianity includes Roman Catholicism and the Protestant reform movements that developed from it beginning in the early sixteenth century. Eastern Christianity includes autonomous faith expressions such as Albanian, Coptic, Greek, Romanian, and Russian Orthodoxy.

### Roman Catholic Christianity

As the church grew in its first several centuries, it quickly developed a hierarchical structure that differentiates it from Judaism. Pronouncements on moral issues come from the top of the church structure, the Papacy, and though individuals might disagree, the pronouncements have a key impact on what the Catholic laity thinks. These pronouncements rely more on biblically based moral theory rather than on analysis of past statements of authoritative individuals.

A full decade before the Harvard Committee convened, Pius XII, leader of the Church from 1939–1958, addressed the International Congress of Anesthesiologists to answer questions raised by one of their members. Included in his response were the moral issues of defining death and withdrawing mechanical support.

In a section titled “When is one ‘dead’? Pius XII makes two key statements. He distinguished between the death of the whole person and the death of organs.

But considerations of a general nature allow us to believe that human life continues for as long as its vital functions—distinguished from the simple life of organs—manifest themselves spontaneously or even with help of artificial processes [31].

He went on to note that:

It remains for the doctor, especially the anesthesiologist, to. . . give a clear and precise definition of “death” and the “moment of death” of a patient who passes away in a state of unconsciousness [31].

These statements were later used to affirm the Catholic position that neurological criteria, determined by the medical community, were appropriate definitions of death. The authors of the Harvard criteria drew on this statement in their report.

John Paul II presided over Roman Catholicism from 1978 to 2005. In his writings and talks, he developed a clear and consistent understanding of determining death that followed from the statements of his predecessors.

In a 2000 address John Paul II noted that “death . . . results from the separation of the life-principle (or soul) from the corporal reality of the person,” acknowledged that this was not observable, and affirmed the use of medical criteria to identify “*the biological signs that a person has indeed died*.” [32] (Italics in the original).

In 2005, the Pontifical Academy of Sciences revisited the issues. Two years later, it published its findings under the title, “Why Brain Death is Valid as a Definition of Death.” The document that resulted opened with the following statement:

Brain death has been a highly important and useful concept for clinical Medicine, but it continues to meet with resistance in certain circles. The reasons for this resistance pose questions for medical neurologists, who are perhaps in the best position to clarify the pitfalls of this controversial issue.

To achieve consistency, an important initial clarification is that brain death is not a synonym for death, does not imply death, or is not equal to death, but ‘is’ death. ([33], p.5)

In this report the Academy noted that cardio-pulmonary criteria had long been the standard for determining that death had occurred, but with the advent of the ventilator and need for organs, medicine developed new criteria. In discussing the Academy findings, John Paul II delineates the Harvard Criteria and subsequent emendations to it in an argument that affirms his acceptance of these criteria when strictly applied. He clearly states that it is appropriate to turn to medical authorities in order to assess the criteria and notes that Harvard Brain Death Criteria (1968) and that the Uniform Determination of Death Act are accepted by all 50 American States, the American Medical Academy and the American Bar Association. Because Papal authority carries significant weight, the roughly 600 Catholic hospitals in the American medical system all adhere to neurological determination of death.

In the past few years, a few Roman Catholic writers have taken issue with the use of neurological criteria writing articles such as “Are Organ Transplants Ever Morally Licit?,” “Brain Death is NOT Death,” and *Finis Vitae* ([34], p.ix). They uniformly oppose the use of neurological criteria and argue instead that cessation of the cardio-pulmonary system is the only licit determination of death. “Neurological criteria are not sufficient for declaration of death when an intact cardio-respiratory system is functioning” [35]. Although this view remains in the minority, it has recently attracted more adherents, though many of those may be influenced by erroneous and misleading statements made in the Terri Schiavo case that she was brain dead [36] and internet sources that make factually false claims like the following, “‘Brain death’ or ‘death by neurological criteria,” is common medical terminology for patients who are said to be in an irreversible coma, sometimes referred to as a ‘persistent vegetative state’ (PVS)” [37].

A recent article released by the Catholic News Agency [38] addresses the concerns of the growing number of opponents to the papal view by rehearsing the tradition on the issue, reviewing the past decisions of the papal authority, demonstrating the widespread agreement with neurological criteria, and pointing out the errors in the cases that are being used to undermine the Church’s position by noting that in those instances individuals either did not meet the criteria in any way or faulty medical practice led to diagnostic errors. This article insists that “death consists in the disintegration of that unitary and integrated whole that is the personal self. It results from the separation of the life-principle (or soul) from the corporal reality of the person.” That disintegration, it argues, is completely evident in the medical criteria used in the determination of brain death [38].

### Withdrawal of mechanical support: Roman Catholicism

Because the official position of the Roman Catholic Church firmly asserts that brain death is death, there is no difficulty in ending the mechanical means that was used to support the patient prior to the determination of death.

The Church uses guidelines to assist in deciding when withdrawing support prior to determining death is a morally fitting decision. To assess the appropriateness of an action in a complex moral situation such as withholding or stopping treatment, Roman Catholicism draws on an ethical system developed by thirteenth century theologian, Thomas Aquinas, and a 16th century lecture on stopping treatment by Francisco diVittoria. Both men provide a method for ethical analysis.

Aquinas’ schematic, called Natural Law, is used to determine how to act morally when both a good and evil consequence might arise from a single action. Four criteria must be met for an action to be considered moral. First, the action must arise from a good will, second, there must be a proportion of good over evil arising from the action, third, the evil may not be directly intended, and finally the evil must not be the means of producing the good. Administering a drug that will provide pain relief to a terminally ill patient (the good) but will also depress the respiratory system and hasten death (the evil) exemplifies double effect. Giving the drug can be considered acceptable if the action is intended to benefit the patient, the lessening of suffering is sufficient to outweigh the shortening of life, the death is not directly intended and the patient is not killed in order to end suffering.

In addition to Natural Law, Roman Catholic medical ethics make use of several principles Francisco DiVittoria set out in his discussion of end of life issues. DiVittorio asked, “What means shall be taken to prolong life when one suffers from a fatal disease?” To this he answers that only those treatments that prolong life for a significant amount of time and do not cause a grave burden for the patient are required. He developed the categories of ordinary and extraordinary means [39]. Ordinary means will prolong a patient’s life and are required; extra-ordinary means, in contrast, prolong death and may be discontinued.

In his 1957 address Pius XII referred to both Natural Law and extraordinary means in his discussion of when treatment can be withdrawn. Following Aquinas, he advises decisions based on the proportion of good over evil in direct relation to the particular circumstances of the individual. Extra-ordinary means, in the face of an incurable illness, are not required. The resulting death from the interruption of mechanical means would be an indirect cause and would satisfy the criteria of “the principle of double effect” [31].

Statements by John Paul II reiterated these standards of care in his 1980 statement, “Euthanasia” and discussed the terms ordinary and extra-ordinary means which he stated had become unclear because the same treatment, a ventilator, for example, would be considered ordinary in one circumstance and extra-ordinary in another. Instead, he uses only the terms proportionate and disproportionate means which can be determined by analyzing particular cases and the types of treatments, costs, physical and moral resources of the patient. He states, “it is permitted, with the patient’s consent, to interrupt these means” [40].

Because of the hierarchical system of Roman Catholicism, statements of the papal authority have significant weight in the Catholic Health Care system. There is one set of directives, for example, for all Catholic health care facilities in the United States. Regulation 33 states, “The well-being of the whole person must be taken into account in deciding about any therapeutic intervention or use of technology Therapeutic procedures that are likely to cause harm can be justified only by a proportionate benefit to the patient [41]. This includes withdrawing enteral or parenteral nutrition in a patient who is dying, but not from someone in a persistent vegetative state.”

### Eastern Orthodox Christianity

The schism that divided Christianity into two main factions began in 1054 over issues that are not germane to this discussion. Contemporary Eastern Orthodoxy differentiates itself from Roman Catholicism in several ways, two are important for the moral issues discussed here. Eastern Orthodoxy does not recognize the Roman Catholic Pope as the supreme authority over all of Christendom. Eastern Orthodoxy is organized into autonomous regional churches. Each regional Church has its own governing body which results in differences across the tradition in a variety of areas including some medical ethical issues. Though it is hierarchical, Orthodox Christianity sometimes cedes the decision making in ethical dilemmas to the individuals in consultation with their spiritual fathers. The decision to use non-abortive contraception, which the Church generally opposes, is left to individuals in consultation with their spiritual father.

Although Eastern Orthodoxy still shares a concern for basing morality on the Bible and authoritative interpretations from the Church Fathers with its Roman counterparts, it does not accept the traditions that developed in Rome after the split. This includes the development of moral theory that began with Thomas Aquinas in the thirteenth century. Eastern Orthodoxy does not believe that moral issues can be understood through either the processes of reason or the use of philosophical analysis.

Writing about moral decision making, Orthodox Priest, Dimitri Cozby, notes that “many issues in this fallen world do not resolve readily into a choice between right and wrong but rather engage us in a struggle to discern the least harmful among various evils.” He goes on to note that “in the Orthodox view, the Fall has corrupted man’s intellect, affecting his ability both to perceive the realities of this world and to deal rationally with those realities.” [42] Because human reason is flawed, Orthodox Christianity, does not have as Tristam Englehardt, philosopher, physician, and convert to this tradition puts it, “moral theology or a moral philosophy” but relies instead on “knowledge born of prayer and illumination by the personal God” [43]. Priest and ethicist Stanley Harakas writes that moral judgments must proceed from harmony with the “mind of the church,” but acknowledges that occasional differences in Orthodox writings on ethics do occur because human reason is flawed [44].

Greek Orthodox bioethicist, John Breck describes what is necessary to do ethics. He says “a scriptural mind” and “a patristic mind” are requisite” and that means “we have to become so steeped in the tradition of our church that our thinking naturally takes its contours.”^h^ The ethicist, priest and layperson all can, in Breck’s view, acquire the “mind of the church through knowledge of Holy Scripture and Holy Tradition” [45]. Philosophical ethics are not sufficient to handle of complex ethical issues because the human mind does not have the requisite knowledge to determine what the consequences of an action will be. That knowledge can only be gained through prayer and the study of scripture and the Church Fathers [44].

Orthodoxy finds the Roman Catholic’s concepts of the principle of double effect and ordinary and extraordinary means to be at times useful but sees that these cannot solve every moral issue. Breck uses the example of a physician treating a terminally ill patient who has assented to a plan to alleviate suffering in a way that will hasten death, Breck notes that by using the principle of double intent, this action would be permissible, because the evil (here the death of the patient) is not technically intended. He believes, however, that it is unreasonable to expect that the doctor will be able to suppress the underlying desire that the patient die so that suffering is permanently relieved; hence the intent actually is to end the life of the patient. Breck along with other Orthodox ethicists believe it morally acceptable to lessen suffering even if the method brings an earlier death when it is done for the benefit of the patient; their disagreement is with the method of arriving at this conclusion [45].

Some writing on behalf of their Church fully accept the idea of brain death. Russian Orthodox Christianity, for one, considers cardio-pulmonary cessation to be a clear indication of death, and goes on to note that the improvement in intensive care technologies “has posed the problem of the verification of the moment of death.” The official pronouncement of the Moscow Patriarchate, Bases (sic) of the Social Concept of the Russian Orthodox Church, notes that new technologies can artificially support organs for a long time, even though the patient is dead. This branch of Orthodoxy cedes the decision of determining death to the physician acknowledging that “this places a qualitatively new responsibility on contemporary medicine” [46]. This branch of Orthodoxy does not take any position on what criteria the physician should use.

Two views present themselves from the Greek Orthodox Church. John Breck finds the medical criteria helpful but not definitive: “If the cerebrum, including the cortex, is permanently destroyed, then we can say that personal identity is lost. While the soul is the life principle of the entire organism, death of the cerebrum indicates that the soul, in liturgical language, has ‘left the body’ and the person as such is dead”. But this writer cautions, “answers regarding appropriate treatment are not written on the wall”. The only way to make a moral decision is through ceaseless intercession and offering the patient to God, “asking for both clarity and charity” in making these decisions [45].

Nikolaos Hatzinikolaou of the Hellenic Center for Biomedical Ethics in Athens, points out that “death can be generally described but not exactly defined, “because along with a biological event it implies an unknown mystery, hence, he adds that “the Orthodox Christian Church avoids clear-cut statements that identify death with the cessation of the brain, cardiac or any other function” [47]. Despite his disagreement with basing determinations of death exclusively on medical criteria, Hatzinikolaou goes on to state:

It seems that brain death will remain open to discussion. However, from a spiritual point of view, this does not create any ethical problems to transplantations. It may be even better for it makes us transcend the scholastic certainty of a clear-cut definition of death and introduces us to the uncertainty of a risky decision. Love cannot be expressed without taking risks! [47]

Though it does not deal specifically with issues of end-of-life issues, the Coptic Orthodox Church states that it “does not have (and actually refuses to canonize) an official position vis-à-vis some controversial issues (e.g. abortion). While the church has clear teachings about such matters (e.g. abortion interferes with God's will), it is the position of the Church that such matters are better resolved on a case-by-case basis by the father of confession, as opposed to having a blanket canon that makes a sin of such practices” [48].

These views are unified in their overarching position that each moral issues must be looked at individually. Indeed, Orthodox moral reasoning can turn to the principle of *Economia*, which allows flexibility where there is a clear cut pronouncement that does not seem to be the right decision in the case at hand. *Economia* in canon law and in ethics authorizes exceptions to the rule without considering the exception either to set a precedent or to abrogate the rule. The justification for applying *Economia* is avoidance of the greater harm that would come from the strict application of the rule.

### Withdrawal of mechanical support: Eastern Orthodox Christianity

Orthodox Catholicism, like its Roman counterpart, accepts the withdrawal of life support systems for those who are dying and quotes the Roman Catholic position on withdrawing treatment. Breck states that “withholding or withdrawing life support conforms thoroughly both to the Hippocratic Oath and to the will of God as we know it from Scripture and the tradition of the Church” because it allows the patient to complete life with peace and dignity. He goes so far as to say that in some situations withdrawing treatment is obligatory ([45]: 143). Hatzinikolaou agrees because “hope in the resurrection is incomparably superior to the desperate struggle for the prolongation of earthly life” ([47]:191).

### Protestant Christianities

Protestantism entered the world religious scene in 1517 when Martin Luther (1483–1546) posted his 95 theses on the door of the Wittenberg Cathedral. Two core principles of this Protestant Reformation were the priesthood of all believers and *Sola Scriptura*, ideas which form the core of many contemporary forms of Protestantism. The term, priesthood of all believers, refers to the belief that there is no separate ethic, responsibility, or ability among the believers, clergy or laity, to make moral determinations and it includes the notion that individuals are responsible for turning only to scripture and not to authority to ascertain the morality of an action. The result of *Sola Scriptura* is that most Protestants will not automatically turn to authoritative figures from the past to make determinations though they certainly consult them. It is just as likely that they will turn to present day religious leaders, science, medicine, or philosophical ethics to inform their moral views when Scripture provides no clear guideline.

In contrast to the Protestant Reformation, the English Reformation which began during the reign of Henry VIII (1491–1547) started as a political dispute in 1529 with religious reforms following especially under Henry’s son Edward VI. The contemporary Anglican Communion bases its decisions on reason, scripture and tradition.

The focus on individual authority in both Reformations quickly led to the splitting of the original reform movements into new forms of Christianity; Anabaptist movements in Germany formed within a decade of Luther’s pronouncements; by the beginning of the 17th the Baptist tradition began among English emigrés living in Holland. The divisions continued; the result is minimally hundreds of variations of Protestant Christianity with new forms regularly emerging.

Many forms of Protestantism do not have official statements on the appropriate criteria to use in making a determination of death.^i^ This includes American Baptists, Jehovah’s Witnesses, Seventh-Day Adventists, Southern Baptist Convention, United Methodists, Unitarian Universalist Association, and the United Church of Christ.^j^ All of these groups explicitly, though briefly, state that they do support living and cadaver donations^k^ and find the withdrawal of mechanical means to be appropriate. For example, the 1992 resolution on Euthanasia from the Southern Baptist Convention states: “Most Christians believe it is morally and biblically acceptable for patients to refuse or withdraw medical treatment when death is imminent and do not categorize such practice as euthanasia or ‘mercy killing.’” This resolution prohibits the withdrawal of artificial hydration and nutrition [49].

Lutheran, Anglican, Presbyterians and churches belonging to the National Association of Evangelicals in America^l^ have more substantial writings on these issues than do the groups listed above.

### The Lutheran churches

The Lutheran tradition comes directly from the reformation in Germany. Today, it is organized into autonomous regional churches. It holds firmly to the original principles of the priesthood of all believers and *Sola Scriptura*. Statements on social/ethical issues produced by the regional groups reflect this since they are designed “to educate and guide members” as they form their own judgments [50]. In 1979, the Lutheran Church Missouri-Synod Report on Euthanasia with Guiding Principles found the “criterion of brain death to” to have “contributed to a more constructive discussion” on determining death because earlier definitions of death that focused on “circulation of blood and stoppage of animal functions” did not duly separate humans from the animal kingdom [51].

Similarly, in 1983, Daniel Lee wrote a study guide to accompany the Lutheran Church in America’s^m^ statement, Death and Dying. Lee wrote,

The whole or total brain definition has the most to recommend it. Unlike the upper or higher brain definition it does not reduce the concept of death to irreversible loss of consciousness. Nor does it violate social sensitivities in the way that the upper or higher brain death definitions does. Unlike the spontaneous heart-lung definition it does not run the risk of declaring death when consciousness is still possible. And unlike the more inclusive heart-lung definition, it does not by implication extend the definition of human life beyond the point where integrated functioning of the organism as a whole is possible [52].

The church also publishes works by individual authors that are designed to foment discussion. The website of the Wisconsin Synod, for example features an article on brain death by Lutheran pastor, James Pope, who responds to a question regarding the validity of the concept of brain death. Pope does not see brain death as death. He writes, “if there truly was brain death, the body would not be cycling and processing the oxygen received through the ventilator. The body would not be staying warm”. Then, Pope refers the questioner to another website that argues that brain death is death [53].

### Withdrawal of mechanical support: Lutheran

All branches of the Lutheran tradition support the right of the individual to make their own decisions which can include withdrawing mechanical support. In 1992 the ECLA wrote that “Patients have a right to refuse unduly burdensome treatments” and though the patient should be the primary decision maker, others directly involved can step in if the person is incapacitated [54]. The Lutheran Church Missouri-Synod agrees that support can be withdrawn but cedes the decision making to physicians [51].

### The Anglican communion

Mid-sixteenth century reforms to the Christian Church in England distinguished it from the church in Rome. Theologian, Richard Hooker, (1554–1600) formulated the view that Scripture, reason and tradition were the legitimate basis for theological and ethical judgments. Provinces and national churches of the Anglican community throughout the world are connected to the Church of England through ecclesiastical structures. Scripture is a key source for ethical analysis, including bio-ethics, and is used in conjunction with tradition, science and philosophical ethics. There are variations across the Anglican Communion on a number of ethical issues. On the two issues under study here, however, there is considerable agreement.

A study guide that was prepared in 1998 for a Canadian task force on end of life issues does not discuss the criteria for death in any detail, but does define it. The study materials that were distributed prior to the final report included the following: “the term brain death relates to the clinical criteria developed to determine that death had occurred in patients on life support. Such patients are dead, and the removal of ‘life support’ simply acknowledges this” [55].

In 2000 the Standing Commission on Human Affairs and Health created an End-of-Life Task Force that published a report and later a book [56]. Neither specifically address the issues centering on the criteria for determining death, but in the section on organ donation, it is clear that the task force agreed with medical criteria for determining death and strongly advised its members to consider living and cadaver organ donation [57].

Similar definitions appear in materials that are being distributed for the next end-of-life task force of death and dying that was formed in October, 2014. Proposed and actual changes to laws regarding physician assisted suicide are among societal factors leading to the need for a new task force. To prepare for the new examinations of the issues, Anglican ethicist, Eric Beresford, has prepared materials for the group that include papers on both sides of the issue and response forms participants are to answer after deliberating over the issues.

### Withdrawal of mechanical support: Anglican

While clear definitions of death are hard to find in official publications, support for the withdrawal of mechanical support is clearly stated in both study guides and publications. Artificial hydration and nutrition are included in the medical interventions which can be withdrawn. Acknowledging that withdrawing treatment may be an agonizing decision for families, various Anglican writings uphold the right of the individual and family to make decisions in these cases and the Church provides resources to their congregants to support those making difficult moral decisions about the use of medical treatment [57].

### Presbyterian churches - USA

Presbyterian beliefs and practices are rooted in the theology of John Calvin (1509–1564) who saw understanding Scripture as central to Christian life. In this tradition, individuals are responsible for cultivating their own spirituality through study and reflection. The Church supports this by providing resources to help individuals understand and assess complex moral situations. Members may find statements issued by the governing body, the General Assembly, helpful, but they are not binding. In 1995 The Congregational Ministries Division produced a study guide In Life and Death We Belong to God: Euthanasia, Assisted Suicide, and End-of-Life Issues, for its members and congregations. It contains reflections on scripture relating to death and dying, articles from philosophical ethics, and an extensive bibliography. This manual states that the definition of death arising from the Harvard ad hoc Committees original document “has been widely accepted as the best objective standard by which to determine death and has been adopted into legislative statues and hospital policies” [58].

### Withdrawal of mechanical support: Presbyterian

In 1988 the General Assembly produced a position paper dealing specifically with questions about withdrawing support. This document begins with a scriptural exploration and notes that while the Bible does not give specific instructions it does “offer principles which can and must guide decisionmaking (*sic*).” These principles include attempting to know the “mind of Christ” which “must be informed by Biblical principles as well as medical facts.” Acknowledging that no one rule can govern every end-of-life moral dilemma, the paper states that:

“Ethical choices may become more clearly evident if the goals of medical care in these situations are, first, to heal or restore and, second, to relieve suffering. It is not the goal of medicine simply to prevent death. Thus, the goal of medical care to relieve suffering remains clear even when healing or restoration is not a realistic hope. This goal is likely to prevent the use of technology that prolongs death and often increases the suffering of the patient” [59].

### National association of Evangelicals in America

Roughly fifty denominations and fellowships belong to this association. They include a number Baptist, Lutheran, Methodist, and Pentecostal expressions that have not joined with the national associations. The Association has created a brief statement dealing with determining death. It notes:

The National Association of evangelicals believes that in cases where extensive brain injury has occurred and there is clear medical indication that the patient has suffered brain death (permanent unconscious state), no medical treatment can reverse the process [60].

In the further discussion, however, it makes the following statement:

Brain death is not the equivalent of a coma. A patient might awaken from a coma, but not from brain death. Removal of any extraordinary life-support system at this time is morally appropriate and allows the dying process to proceed [60].

Like writings found in a few other groups, this short treatise argues on the one hand that brain death is death but then adds that withdrawing “life support” allows the dying process to proceed.

### Withdrawal of mechanical support before death has been determined: national association of Evangelicals

In its statement on Euthanasia, the National Association sees the withdrawal of life-support systems to be appropriate when this treatment serves “only to prolong the dying process” [60].

### Islam

Islam appeared on the religious scene in the seventh century through a series of prophetic visions received by Muhammad and eventually codified in the Qur’an. Islam traces its roots to Abraham and accepts that Moses and Jesus were prophets. However Muhammad is understood to be the final prophet and the Qur'an the final revelation of God.^n^ In Islam, God created humans from a single *nafs* (soul or spirit) (7:189) and are variously described as one unified being, or a dualistic being made up of two components, body and soul. That Islam is rooted in both Jewish and Christian thought and influenced by Greek philosophy is clear from these competing and often unclear views.

To determine the morality of action, Islam draws first on the Qur’an and Sunnah^o^ (actions and sayings of the Muhammad as recorded in the Hadith). These Islamic texts spend considerable time talking about death and afterlife, but not the criteria for declaring death. Consequently, contemporary moral discussion will also include interpretation of previous sources to determine what is morally fitting, *halal*, and conforms to the Islamic moral and legal code, *shari’a*. If texts are silent on an issue, Islamic jurists create *fiqh*, *l*egal interpretations, using principles such as the dominant probability of good or harm resulting from an act and potential societal benefit to make ethical decisions ([61], p.317). As in Judaism, the interpretations of text and rational arguments can vary widely since there is no one person who is vested with interpretation for the group as a whole. On occasion, when national councils are tasked with determining the morality of a contemporary moral issue, those who dissent will simply convene a new council and produce an alternative ruling.

Before the advent of mechanical life support measures, there was no disagreement between physicians and clerics about the definition of death. Because of Islam’s profound respect for medicine and science, believed to be mandated by the Qur’an, clerics deferred to physicians on bioethical issues. Hence, Muslim physicians have served a key role in applying tradition to developing medical situations. When physicians wrote in support of brain death, they relied on two Islamic traditions. The first was as an ancient ruling that if the king can no longer use his mind, a new king can be crowned. Since only a deceased king can be replaced, this ruling is used to justify applying brain death criteria to a potential donor. The second, more common discussion, looks to rules hunters use to determine whether an animal is dying or has died. If an animal is to be consumed by humans, Islam requires that it be slaughtered in a specific, ritual way. If a hunted animal dies before the ritual slaughter then its meat is not *halal* and cannot be eaten. Because the Qur’an considers a decapitated animal to be dead, it cannot be ritually slaughtered and cannot be eaten. This idea is then applied to the determination of death in humans to argue that brain death is an appropriate vehicle for assessing that death has occurred ([62], p.152-153).

While there is widespread support from Islamic physicians for using neurological criteria, religious scholars overwhelmingly dispute it.

Beginning in the 1980’s, several Muslim philosophers began attempting to define life as embodied consciousness, a definition they then used to justify using neurological criteria as an indication that consciousness has left the body, which, they believe, coincides with brain death ([5], p.19). Most Muslim jurists, however, believe this cannot be ascertained medically and as a result find cardio-respiratory death the only suitable determination ([62], p.163). Jordanian jurist Muhammad Na’im Yasin dissents from the typical clerical view. He does not believe any textual ordinance is supported by the *Qur’an* or *Sunnah* so brain death is not regulated by any legal precedent. He cedes to physicians the ability to make determinations and argues that the medical evidence for brain death shows a “dominant probability” that the patient has entered the “phase of death.” He argues that the “human soul in its presence or absence remains linked to the brain” ([63], p.317-318). For the most part, however, opinions throughout the Muslim world fall into the three categories: those who oppose neurological criteria, those who accept them, and a third group that technically accepts neurological criteria while acting in a way that belies acceptance of those criteria. A summary of recent judicial decisions exemplifies the varied views.

In Amman (1985), Islamic scholars and medical experts attempted to come to agreement on the criteria for determining death. No resolution was reached during the first session. The next year the group produced its “Resolution of the Council of Islamic Jurisprudence on Resuscitation Apparatus, Amman, 1986.” The resolution reads as most do in Islamic nations. It has provisions for both brain death and cardio-respiratory death satisfying both the clerics and scientists.

A person is pronounced legally dead and consequently, all dispositions of the Islamic law in case of death apply if one of the two following conditions has been established:

1. There is total cessation of cardiac and respiratory functions, and doctors have ruled that such cessation is irreversible.
2. There is total cessation of all cerebral functions and experienced specialized doctors have ruled that such cessation is irreversible and the brain has started to disintegrate [63].

In Kuwait, two councils on jurisprudence produced opposite conclusions within a short period of time – one arguing that the presence of a heartbeat always indicated that the patient was alive, one claiming neurological criteria trumped the beating heart ([5], p.20). The Ayatollah Khomeini, spiritual leader of Iran, pronounced as morally acceptable the use of brain death criteria only to have that rejected by Iranian Islamic jurists ([62], p.164). Perhaps the most conflicted ruling comes from the Islamic Juridical Council who determined that an individual who met the neurological criteria for brain death was biologically dead but only became legally dead when artificially supported breathing breathing stops completely ([62], p265-266).^p^

Several scholars contend that confusion results because in the past physicians and governments acted as primary decision makers in medical situations and “. . . the necessary debate involving religious and legal communities on the issue of when to call patients dead” had not occurred [64]. In the early stages of the debate, Islamic jurists deferred to physicians and generally did not present arguments based on Islamic understanding of either the nature of the human being or on criteria used to determine that death had occurred. Omar Haque contends that the disconnection between “the popular and religious sentiment and emerging scientific practice among physicians” ([5], p.3) has its roots in this deference and lack of education about the issues public debate would engender.

What appears to be the case in Islam is similar to Christianity, namely there is only partial understanding of the actual criteria. In one survey of fifty Islamic jurists, 90% indicated that they did not agree that brain death equaled death. After an explanation of the criteria used, seven emended their view to indicate the appropriateness of brain death ([5], p.28). In this situation, when some individuals were better informed on the details of brain death and its differentiation from conditions such as persistent vegetative state, they were more inclined to accept neurological criteria.

### Withdrawal of mechanical support before death has been determined: Islam

Though the issue of how to determine if someone has died remains contentious, there is little disagreement on withdrawing futile treatment. The physician and family are charged with making this decision. Factors that can be considered include the benefit to be derived from the treatment, the burden to the patient and the burden to the family.

### Indigenous traditions

Also called aboriginal, primal or archaic (from the Greek *arché*, original) religions, indigenous traditions are found throughout the contemporary world from the North American Cherokee to the Ghanaian Ashanti and everywhere in between. Although each form has unique aspects, these traditions share features that cut across regional boundaries. These include an animistic world view, concern for universal harmony, centeredness in nature and place, and right practice.

Animistic traditions have several features that have bearing on the discussion at hand. In Animism, humans have both a physical and a spiritual component. At death, the spirit, often called an ancestor, will both abide in another world and be present in this one. Respect for those who have died is a hallmark of these traditions; some include ancestor worship. Seeing themselves as part of a whole living world, indigenous traditions often value the land and place. Sacred rocks, mountains and religious artifacts made of clay and stone connect people to the land. The correct enactment of life stage rituals protects the entire community and keeps evil forces at bay. Disruption to harmony can arise from an individual breaking a taboo–whether intended or not–or from improper ritual performance. The orientation and goal of moral reasoning in these traditions is maintaining harmony. Stories about the ancestors and listening to their voices and those of spirit beings and animal helpers provide the moral framework for decision-making [65]. Often, the entire community involves itself in determining how to act. At other times, community elders will decide for the group. Each moral decision is seen to affect the entire community. Failure to follow the proper procedures in caring for the dead, for example, will bring harm to the deceased and the community.

For most indigenous traditions including those in China and Japan which are discussed below, mentioning death is taboo; a common view is that talking about death will invite it [66]. Most indigenous groups share a focus on the taboos, proper rituals and the location of death, which should be outdoors, on the ground or in a specially constructed hut. Indigenous people around the world have resisted medical intervention in death. This is especially true in areas where there is a “culture of colonization,” that has produced suspicion and outright mistrust of institutions and their bureaucracies. . .“In addition, the culture heritage of nature based healing sometimes conflicts with biomedical interventions and technological solutions to health problems [67]. ^q^These reasons, combined with a desire to choose the location of death has several implications for understanding indigenous attitudes toward death. First, very little information is available because of a reluctance both to discuss death in general and to “talk about it with non-Indians” ([68], p.177). Second, the desire to die in a specific location--at home or outdoors, for example, means that hospital deaths are often avoided. For some groups, the place where death occurs becomes dangerous and community members will avoid it for varying periods of time making hospital deaths particularly problematic. Third is the view that medical intervention at death should be limited ([69], p.121).

### Withdrawal of mechanical support before death has been determined: Indigenous traditions

Attitudes toward withdrawing mechanical support in China and Japan are discussed below. In the Americas and Australia there is information from a variety of indigenous expressions [70] that does point to reluctance to accept medical treatment at the end of life and a desire to die in the home area [71]. These two factors can be related especially when the reservation is located at some distance from medical facilities; dying in hospital would remove the individual from their community support system. In addition, a typical, though not universal view, is that individuals should die in the land where they are born, otherwise their spirit will wander aimlessly ([72], p. 429). One subject of an interview commented that when they are dying, “people should go home. Because their spirit will be lost, yeah, looking for home.”^r^ ([73], p. 264–268).

### Eastern religions

Eastern Religions include Hinduism, many varieties of Buddhism as well as Confucianism and Taoism found in China and Japan. Many Eastern religions are layered onto pre-existing, indigenous traditions.

### Hinduism

Hinduism is the name Persians and Greeks gave to the grouping of religions found in India and presents itself in varieties that have regional and cultural differences. In general, Hinduism believes the current body is not the true self—rather the body is like a coat that is shed when it wears out. Underneath the coat is the self, Atman, which moves from life to life. The actions you do in this life, Karma, will determine the form that your body takes in the next life. The ultimate goal is to gain permanent release, *moksha*, from the cycle of death and rebirth and experience oneness with *Brahman* (God). Intermediate goals vary from group to group but many include possible existence in paradise which, though temporary, will be blissful, or birth in a better life station which will increase the chance of gaining permanent release.

Hinduism characterizes itself as a way of life rather than as one religious tradition and centers on practice, specifically doing what is right, or *dharma*. To assess the correct action in a moral issue, Hindus will consult scriptures, reflect on tradition, follow examples of gurus, and consider their duty as it applies to their gender, caste and life station. The overriding issue that determines what action to follow is the goal of *moksha*, liberation from the cycle of death and rebirth ([73], p.162). When dealing with issues regarding death, however, many Hindus are uncomfortable with medical determinations of death [74]. Although India passed a resolution in 1994 that included provisions accepting brain death criteria, and several sources state that Hindus accept neurological criteria [75], internet and library searches turn up few discussions of the issues surrounding medical definitions of death. Instead discourse on death typically focuses on what constitutes a good or bad death in religious and next life terms. A good death, essential for both the dying person and the family, occurs when the individual is properly prepared to die, the astrological signs are right, and the proper rituals are performed. Experiencing either liberation or a good rebirth requires a good death. A bad death will have permanent ramifications in subsequent lives; the deceased may be reborn in a lesser station or wander unable to be reborn but not liberated. There will be unfortunate consequences for the family–e.g., bad luck, nightmares and infertility—whose members will have to perform retributive rituals for years to offset the bad death. Sudden death, death with excessive bodily fluids, or a death accompanied by poorly performed rituals constitutes bad death ([76], p.683).

The place of death also matters. Dying in the holy city of Benares near the Ganges is preferred, but if that is not possible individuals will be lowered to the floor to avoid the area between the ceiling and floor which is filled with turmoil.^s^ Water from the Ganges is sometimes placed under the patient and is usually given by mouth. The focus of the individual must remain on a religious thought ([77], p.117), because “The dying should always remember that the place where one will reincarnate is the place that he is thinking about prior to death” [78]. Some people chant the name of their deity, others chant passages from the *Bhagavad Gita*, and family members place a light near the head of their loved one. Interrupting this process can have consequences that will continue eternally since it will affect all future births.

Most people do not die in hospital in India; rather they are sent home when death approaches ([79], p0132). This tradition, along with Hinduism’s focus on right practice, contribute to the scarcity of religious discussion about issues such as determining death.

In a study of Hindu immigrants to North America, Kyoko Murato outlines the adaptations the community has made to death rituals. In North America, people do die in hospitals and the Hindu community has adjusted to that. Murato also notes that in urban areas in India, there are gradual changes in practice ([79], p125). Water from the Ganges is brought to the hospital room and the practice of moving the patient to the floor is abandoned.

### Withdrawing treatment before death has been determined: Hinduism

Hindu concern for both interfering with the timing of death and allowing the individual to focus correctly shows itself directly in attitudes toward suspending treatment, especially mechanical means of life support. Twentieth century saint and spiritual master, Sivaya Subramuniysawami (Gurudeva) (1927–2001) notes:

To make heroic medical attempts that interfere with the process of the patient’s departure is a grave responsibility, similar to not letting a traveler board a plane flight he has a reservation for, to keep him stranded in the airport with a profusion of tears and useless conversation. To prolong life in the debilitated physical body past the point that the natural will of the person has sustained is to incarcerate, to jail, to place that person in prison. The prison is the hospital. The guards are the life-support machines and the tranquilizing drugs [80].

This attitude is shared by Hindu people living in India and those who have migrated to new lands; families will attempt to avoid artificial life support or terminate it as soon as its futility is evident. Moreover, suspending artificial nutrition and hydration is sometimes supported because there will be fewer bodily fluids and the soul can more easily leave the body.

### Buddhism

Founded in the 6th century b.c.e by Siddhartha Gautama, Buddhism is the largest of several religions that appeared in India at that time. It quickly became popular, moved throughout Asia and was able to meld itself to the pre-existing cultures of the locations in which it found itself. For example, Buddhism is practiced alongside indigenous traditions, such as *feng-shui* in China and Shinto in Japan, and earlier formal religious and philosophical systems, Confucianism and Taoism. Like Hinduism, Buddhism believes in the cycle of death and rebirth, *samsara*,and proffers a way to liberation, *Nirvana*. There are two main types of Buddhism, *Theravada* and *Mahayana*; there are only slight variations between these two groups on the issues of determining death.

Key sources for ethical guidance include the Buddha whose life embodied truth and the ability to be liberated from the cycle of births, *samsara*, the Buddha’s teaching, *Dhamma*, and the community of advanced practitioners, the *Sangha*. Lay people will rely on monks and nuns for guidance in making moral decisions, but they are not obligated to follow their advice. Some forms of Buddhism have a key figure such as the Dalai Lama, but there are few authoritarian pronouncements that must be observed by all followers since the Buddha emphasized the necessity of finding one’s own path.

In Buddhism, death is distinguished from life by the absence of three things, vitality, heat and consciousness. Two western Buddhist scholars, one traditional physician-monk from Thailand, and the president of the lay Buddhist group, Soka Gakkai International, are the only Buddhists who have argued publically that brain death criteria are consistent with the Buddhist understanding of death. Scholar Damien Keown, saw the category of vitality and heat corresponding to bodily metabolic processes that generate heat and claimed that Buddhist texts support the idea that the brain is the source of integrating consciousness.^t^ Soka Gakkai president Daisaku Ikeda, has written that brain death appears to meet the criteria for death in Buddhism, but true to Buddhist form, he indicates that individuals have to come to own conclusions ([81], p.61).

Keown has, however, recently changed his view and now agrees with most of Buddhism that the loss of bodily heat is the only reliable indicator of death ([3], p.9). Keown and others argue that consciousness, called “the subtle body” continues to exist in a person whose heart and respiration have stopped [4].^u^ Tibetan Buddhists claim that it takes a minimum of three days for the subtle body to depart [82-84]. Adherents to Pure Land Buddhism of China and Japan believe this happens over a period of twelve to twenty-four hours. Scientific tests are not adequate, most Buddhists argue, “because they may not be able to detect existing states of consciousness which may be in the [non-beating] heart, preparing for death” ([85], p.306). Disrupting the body prior to actual death will interfere with rebirth or attaining release from *samsara*. For Buddhism, the ideal death occurs “when there is no anxiety and the person is in a conscious, calm, uplifted state” ([85], p.292).^v^

A brief look at Buddhism in several locations illustrates how these general features operate and how they merge with other religions.

### Chinese traditions

In China, Buddhism is present alongside indigenous practices such as devotion to ancestors, and the philosophical/ethical systems of Confucianism, and Taoism. Buddhism arrived in China in the first century c.e. and by the eighth century had its own distinctive forms called Pure Land and Chan (Zen in Japan). Pure Land Buddhism is the most popular form of Buddhism in China; adherents are devoted to a Buddha called, Amitabha, who presides over a heavenly place called the Pure Land. People who believe in him pray to him and meditate on him so that they will join him in his paradise when they die. The Pure Land is a desirable spot described as a fragrant paradise with hundreds of thousands of colors. It is filled with precious things. When people arrive, they can do whatever they want [83].^w^ Chinese understandings of death come from a blend these traditions.

From indigenous roots China takes reluctance to discuss death and veneration of ancestors. From Confucianism it takes proper practices and filial piety, and from Taoism, a focus on Nature, prolonging life and the idea of life force that pervades the universe. To ensure the passage from death to the status of ancestor, complex rituals are required and begin immediately when the life force is gone. Failure to perform appropriate rituals will affect the deceased and the family ([86], p.160). The filial obligations that arise from Confucianism may include children protecting parents from the anxiety of a poor prognosis by withholding information and by using whatever means necessary to prolong their parents’ lives as long as possible ([87], p.41).

Death occurs when the life force (*Chi*) leaves the body. *Chi*, a concept in both Taoism and Chan Buddhism, is not located in any one organ but is physically diffused throughout the body. Neurological criteria for determining death are not easily reconciled with the idea of *chi*. Although death practices are changing in China, the idea of a good death occurring at home, in the main hall in the presence of ancestor tablets is still cherished. Proper place of death helps the deceased attain the status of ancestor and ensures harmony ([86], p.156-157).

Pure Land Buddhism is layered onto and practiced alongside these ideas. Dying at home is preferred. To ensure a good death, the dying person focuses on Amitabha by chanting, *Amituofo*. Family will gather in the patient’s room to help the individual chant. Pure Land Buddhists believe the subtle body leaves the body over a twelve to twenty-four hour time period after respiration ceases. During this time the patient can still feel pain and sadness [86]. Specific rituals will assist the deceased in a safe arrival in the Pure Land. If the process of dying is interrupted, the newly deceased person, ancestors already in the Pure Land, and the current family will suffer. Pure Land adherents prefer not to start artificial means of life support but treatment may be withdrawn if the family feels its obligations to the individual have been met.

Not everyone in China follows all these practices, to be sure, and there is an emerging view that “some traditional views of life and death have become cultural obstacles blocking scientific approaches to the dying process” ([88], p.36). In response to attempts to manage death medically, Pure Land groups have created ‘Living Wills’ that direct relatives to bring individuals home to die and instruct them in proper ritual performance [89].

### Japanese traditions

Taoism, Confucianism and Buddhism migrated to Japan and became part of the Japanese religious landscape which also includes the indigenous Japanese religion, Shinto, the way of the Gods. Widely practiced, Shinto is an optimistic religion that focuses on life, purity, and seasonal rituals [90]. Reverence for nature and what is natural are hallmarks of Shinto. Death is a source of impurity in the Shinto tradition; there are no Shinto funerals or cemeteries. Shinto priests will use the rituals of Buddhism or Taoism when performing funeral rituals.

In Japan, most people die in hospital; the medical infrastructure accommodates itself to death rituals by providing a space in the hospital where the family and hospital staff gather with the newly deceased for ritual practice. Determining death through neurological criteria has provoked serious controversy in Japan. Although criteria for brain death were legislated in 1997,^x^ medical and lay individuals routinely state their discomfort with these criteria, saying that brain death is “unnatural,” “too unnatural to be called death,” “going against natural science” [91]. John McConnell locates the source of this discomfort in the conflict between the neurological definitions of death and the basic goals of Shinto, “preservation of life, promotion of good health, and harmonization with nature in accordance with the way of *kami*” (87], p.322). Shinto followers, he notes, “believe that to declare death while the heart is still beating is premature, as well as unnatural; again, nature is paramount in their beliefs.” This, he argues, is the reason that a society that accepts medical intervention in much of life resists it in death ([90], p.324). Helen Hardacre has written a nuanced article on Japanese Buddhist and Shinto responses to attempts to define death medically. She notes that the Buddhist principle of *engi*, in which the individual is always becoming, is difficult to reconcile with the death of any particular organ or a particular moment in time [91].

### Tibetan traditions

Buddhism entered Tibet from India,^y^ and blended with Hinduism, Pure Land Buddhism and Tibet’s indigenous Bön tradition. Bön includes shamanic belief and practices to banish demons and gain favor from protective divinities and rituals concerning the transmigration of souls after death. A complex system for understanding and negotiating the stages of dying developed from the combination of these religious traditions ([4], p.22). The text, known in English as The Tibetan Book of the Dead, is read at the bedside of the dying to help them reach the goal of the Clear Light, a state similar to *Nirvana*.^z^ Reading continues for three days after the determination of vitality, heat and consciousness because the subtle body has not yet left the physical body in its journey to its next life. The dying process takes a total of forty-nine days after respiration stops [81]. This practice can be incorporated with Pure Land practices for the less spiritually adept who may not have the expertise to enter the Clear Light but have the potential to reach an area where they can improve their chances in their next life.

In her influential book, Buddhism, Bioethics, and Death, Karma Lekshe Tsomo notes that in Tibetan Buddhism there are four ways of determining death. The first requires consultation with a lama who through divination or meditative processes determines whether the subtle consciousness has left the body; the second, third, and fourth depend on empirical observations of the body and include seeing a drop of mucus on the nostrils, determining the loss of body heat, and witnessing the beginning of physical decomposition ([4], p.219). “The critical point for Buddhist,” she writes, “is whether the subtle consciousness is still present in or around the body, though this is not easy to determine.” Tsomo notes that for many forms of Buddhism disturbing or disposing of the body before it is completely clear that the subtle consciousness has departed is “potentially damaging and can lead to fear, attachment and unfortunate migrations” ([4], p.218).

Tsomo recommends not beginning mechanical support at the end of life, since the discomfort it might causes would interfere with the calmness essential to a good death. Once mechanical support is started, however, Tsomo cautions that it should not be discontinued until it can be determined that the subtle consciousness has left the body through the methods mentioned above. She notes that common methods of assessing the presence or absence of ordinary consciousness may be able to measure brain function, but “current scientific instruments are still unable to detect or measure the very subtle consciousness that is thought to continue from one life to the next.” She goes on to state that “current definitions of death that are linked to brain stem function are inadequate to determine the viability of human life” ([4], 220)^aa^.

### Western Buddhism

Over the past several centuries various forms of Buddhism have intersected with the West in a variety of ways, including Colonialism and War, but philosophical and religious interest began in earnest at the beginning of the nineteenth century. At first, “Buddhism was not exported from abroad by Asian emissaries; it was imported from within by European orientalists” who were primarily philosophers and philologists who founded several Buddhist societies. These emphasized the rational basis for Buddhist belief ([92]:82). By the 1970’s several forms of Buddhism become popular in North America and Europe when monks exported their traditions. ([92]:595).

These include Soka Gakki International, which is discussed above, Zen, and a new Western form of Tibetan Buddhism, Shambhala.

Scholars distinguish three ways in which practitioners relate to Western Buddhism. The first is practiced by individuals whose ancestors emigrated from areas where Buddhism was the main tradition. Jōdo Shinshu Buddhist Churches of America, for example, founded in the United States in the nineteenth century is home to Buddhists of Japanese descent. A second type, “ethnic Buddhism, is found among newer immigrants, exiles, and refugees who have come to the United States in the past thirty years from South, Southeast, Central, and East Asian countries,” and a third, often called ‘convert Buddhism,’ refers to those who have turned to Buddhism since the 1960’s ([93]:xix). ^bb^Since the first two types are similar on the issues at hand to the traditions in the countries in which they originated, this section will focus on two forms of convert Buddhism popular in the West, Zen Buddhism from Japan, and Shambhala, a new form of Tibetan Buddhism.

Western convert types of Buddhism differentiate themselves by focusing more on meditation and less on ritual, emphasizing lay practice, and following ‘engaged Buddhism’ which is the “broadening of spiritual practice to include both family and community and the social and environmental concerns of the broader world” ([94]:71). In addition, they draw on a wide variety of material from other faith traditions, science and philosophy.

### Western Zen

After World War II, clerics who were dissatisfied with Zen institutions in Japan began to see the United States as a location for the revitalization of Zen that would be “unencumbered by its institutional history”. By the end of the 1960’s Zen had become the most prominent form of Western Buddhism ([95]:112).

Western and Japanese Zen differ in a number of ways. There are two forms of Zen in Japan, but in the West they have been merged. Japanese Zen is monastic; Western Zen is a lay movement. Western convert Zen does not carry with it Taoist, Shinto and indigenous practices such as ancestor worship.

Western Zen Centers support hospices to provide compassionate care for the dying; Zen practitioners and teachers write considerably about caring for the dying but very little about clinical definitions of death and withdrawal mechanical. The focus, when discussing death, is on a good death, which is an aware death. The goal is to avoid a death where the patient is not conscious when the heart stops beating [96].

In those few places where clinical issues are raised, Western Zen like all other forms of Buddhism, believes the process of dying begins with the cessation of the heart and lungs and ends some time later, often three days, when the subtle consciousness leaves the body [97]. ^cc^Neither cardio-pulmonary cessation nor brain death criteria are accepted in Zen as definitions of what might be called ultimate death though they are seen as definitions of clinical death. In an article on transplants, Casey Frank writes, “it is generally believed that the circumstances of clinical death and the period following it, before the consciousness is released, are critical in helping to determine one’s rebirth.” Frank notes that two Zen Masters, Sheng-Yen and Tangen Harada Roshi believe an individual can overcome the difficulties of disturbing what they see as someone in the process of dying during organ removal. Others argue that it is better to avoid organ donation in case the difficulties cannot be overcome [98].

Withdrawing treatment from an unconscious, dying patient presents issues similar to those of organ donation. The interruption of the dying process can produce serious, unwanted consequences in rebirth [99].

### Shambhala international

Tibetan monk Chögyam Trungpa developed what is now called Shambhala in North America in the 1970’s. Trungpa’s goal was to produce a non-monastic form of Buddhism “free from the cultural trappings of Tibet” [99:75]. Belief in demons and rituals to placate them that are common in Tibetan indigenous religions are not part of this Western form. Shambhala adherents blend their own pre-existing ideas, such as ideas about the nature of body and soul, with their Buddhist views. Shambhala is eclectic and while rooted in Tibetan Buddhism it draws on other Eastern traditions religion as well [100]. The differences between Buddhism in Tibet and Shambhala in the West result in some variations in both thinking and practice.

Blending existing ideas, incorporating the views of other faith expressions, philosophy and science, and making the religion distinctly your own is characteristic of adherents in this group. The Auckland Shambhala Center in New Zealand states: “A unique quality of the Buddhist teachings is that they can be expressed through existing cultural norms, making use of them rather than destroying or replacing them. This allows many westerners to practice Buddhism today without renouncing their cultural heritage or radically changing their lifestyles” [101].

In his book, Preparing to Die, American Shambhala teacher, Andrew Holecek outlines the basic features of Shambhala’s interpretations of Tibetan Buddhism and draws heavily on the Tibetan Book of the Dead as one would expect. But his work takes several decidedly Western turns. He opens his book with the statement, “Don’t measure your death against any other, and don’t feel you have to die a certain way. Let your life and your death be your own. There are certain things in life we just do our own way” ([101]:4). Focusing on the individual and allowing them to choose their own path is typical of what several scholars, beginning with Martin Baumann, refer to as “Protestant Buddhism” because it cedes authority to the lay individual and focuses heavily on self-education [102].

Holecek, who writes for the Shambhala organization, views the practices of the Tibetan Book of the Dead as the most valuable in preparing for death. He carefully explains the ritual practices relating to the deceased and cautions against moving the body until three days after the heart has stopped beating. But in a major departure from Tibetan understanding and practice, he adds, “If the dead person is an organ donor, the generosity of offering their body supersedes these instructions. Organ harvesting should begin immediately.” Rather than seeing the process of dying solely in the terms explained in Tibetan Buddhism, he describes clinical death “as either (1) the irreversible cessation of all the functions of the brain, including the brain stem, and (2) as an irreversible cessation of circulation, heartbeat, and breathing”. Holecek and Shambhala as an institution, in sharp contrast to the majority of non-Western Buddhists, believe that the dying individual can overcome the difficulties in reaching the appropriate afterlife that interrupting the dying process would produce [101]:164).

Although the majority of Buddhist writing on withdrawing treatment determines that it will have harmful consequences for both the dying individual and the person who actively participates in this process, Shambhala has a nuanced view that shows Western influence. Actively killing someone, as in most traditions, is wrong because the karmic effects, even if the intent is good, will be eternal and negative. Removing life support systems, which Shambhala calls passive euthanasia, “only has a soft karmic effect that can easily be overcome”. Holecek writes, “There comes a point when there’s no need to prolong life and incur unnecessary emotional and financial burdens” ([101]:264).

## Conclusion

Patterns emerge in the comparative study of religious perspectives on death. Western traditions show their rootedness in Judaism in their understanding of the human individual as a finite, singular creation. Although the many branches of Western religions do not agree on precisely how to determine death, they are all able to locate a moment of death in the body. In Eastern traditions personhood is not defined in physical terms. Moreover, the influence of indigenous systems on the religions of Hinduism and Buddhism is significant. From prescribing the location of death, to resisting medical intervention and definitions of death, Hinduism and Buddhism in their many forms, echo these indigenous traditions. Adding to the complexity for these two traditions is the idea that death is a process that continues after the body has met most empirical criteria for determining death. For Hinduism and Buddhism, the cessation of heart, brain and lung function is the beginning of the process of dying—not the end.

Although not all members of a given tradition will believe or practice in the same way, they are often guided by the religious perspectives which surround them. In times of crises, and the death of a loved one is surely such an event, even the agnostic might summon long forgotten symbols, rituals and prayers and draw comfort from them.

In his book, The Sacred Canopy, sociologist of religion Peter Berger wrote, “The power of religion depends, in the last resort, upon the credibility of the banners it puts in the hands of” its followers “as they stand before death, or more accurately, as they walk inevitably toward it” ([103], p.115). In the face of death, religious systems have provided mechanisms for coping with all the areas of life into which the death of a loved one intrudes—from prescriptions and proscriptions on handling the body, to ritual obligations that provide concrete actions the family can perform on behalf of the deceased, to comforting visions of afterlives. The struggle with medical definitions of death on the part of many world religions comes, in no small measure, because of the symbol systems and the ways in which they function to keep anomie at bay.^dd^

## Endnotes

^a^This comparative exploration is narrowly focused on which criteria different religions find appropriate when determining death. It does not include a discussion of patients in a persistent vegetative state; no religious tradition considers such a patient dead.

^b^In non-Western traditions, the absence of a heartbeat and/ or respiration would be a signal that the process of death was beginning; it would not indicate that death had occurred.

^c^Rabbi and physician, Moses Feinstein initially opposed the definition of death developed by the Harvard criteria; later he came to embrace it. His views are shared by others including Gedalia Schwartz, Moshe Tendle and the chief rabbinate of Israel.

^d^Bleich is joined in his perspective by other revered Rabbis including Hershel Schachter and Mordechai Willig.

^e^The italicized section is Rabbi Moses Isserles’ gloss on Joseph Karo, *Shulhan Arukh, Yoreh Deah 339* that was completed in 1563.

^g^See for example, Mackler A: *Life and Death Responsibilities in Jewish Medical Ethics*. New York: The Jewish Theological Seminary of America; 2000.

^h^ For the bioethicist, he adds knowledge of science.

^i^The Church of Christ Scientist, (Christian Science), was founded by Mary Baker Eddy in 1879. As an institution, Christian Science does not approve of any medical intervention, hence it has no pronouncements on moral issues that involve medical treatment. Individual Christian Scientists are free to make their own decisions.

^j^Were there church pronouncements, they would not be binding on the members of these groups except in the case of Jehovah’s Witnesses. Blood transfusions are specifically and absolutely prohibited because of what this group sees as a clear Biblical mandate against them. Partaking of a transfusion will have, in their view, eternal consequences and will lead to an individual being disfellowshipped from their organization.

^k^Jehovah’s Witnesses are not allowed to receive bone marrow transplants because of the possibility of transplanting blood. Transplant surgery from living or cadaver donation is allowed if the surgery is bloodless. Organ donation is encouraged.

^l^This web-site of this Association lists the following members: Advent Christian General Conference, Assemblies of God, Baptist General Conference, The Brethren Church (Ashland, Ohio), Brethren in Christ Church, Christian & Missionary Alliance, Christian Catholic Church (Evangelical Protestant), Christian Church of North America, Christian Reformed Church in North America, Christian Union, Church of God (Cleveland, Tennessee), Church of God, Mountain Assembly, Inc., The Church of the Nazarene, Church of the United Brethren in Christ, Churches of Christ in Christian Union, Conservative Baptist Association, Conservative Congregational Christian Conference, Conservative Lutheran Association, Elim Fellowship, Evangelical Church of North America, Evangelical Congregational Church, Evangelical Free Church of America, Evangelical Friends International of North America, Evangelical Mennonite Church, Evangelical Methodist Church, Evangelical Presbyterian Church, Evangelical Missionary Fellowship, Fellowship of Evangelical Bible Church, Fire Baptized Holiness Church of God of the Americas, Free Methodist Church of North America, General Association of General Baptists, International Church of the Foursquare Gospel, International Pentecostal Church of Christ, International Pentecostal Holiness Church, Mennonite Brethren Churches, USA, Midwest Congregational Christian Fellowship, Missionary Church, Inc., Open Bible Standard Churches, Pentecostal Church of God, Pentecostal Free Will Baptist Church, Inc., Presbyterian Church in America, Primitive Methodist Church, USA, Reformed Episcopal Church, Reformed Presbyterian Church of North America, The Salvation Army, Synod of Mid-America (Reformed Church in America), and the Wesleyan Church.

^m^In 1988, The Lutheran Church in America merged with the American Lutheran Church and the Association of American Lutheran Churches to form the Evangelical Lutheran Church in America. Together with the Lutheran Church Missouri-Synod and the Wisconsin Synod, they comprise the largest three forms of Lutheranism in the United States.

^n^The split between the two main divisions of Islam, the Shiite and Sunni occurred shortly after Islam was founded. Though these two groups can diverge widely on many theological and practical issues, they do not differ in general on the issue at hand despite the rift between them.

^o^*Hadith* is the narration of the teaching of the prophet transmitted by those who heard him. The *Sunnah* is the code of practices taught by the Prophet.

^p^Thus a brain dead patient can inherit and the spouse is not widowed.

^q^The authors note that individuals who are less traditional will have views that are more moderate.

^r^Home in this context seems to mean variously, the location of the family unit and the actual dwelling place. See also [79] 429-431.

^s^Other reasons are sometimes given for this; the practice varies little however.

^t^See [80] for a discussion of Bhikkhu Mettananda and L.H. Van Loon. See also [83].

^u^See also [84].

^v^Buddhists note that consciousness may not be observable and recount stories of the Buddha and saints who were mistakenly declared dead when they were in a meditative trance. See also [3].

^w^Chan Buddhism developed, in part, because some Buddhists believed Pure Land to be overly simplistic. Chan is not focused on a heavenly paradise but is a meditative school focusing on developing the individual self. Pure Land Buddhism is highly popular in China; Chan appeals more to people in the West.

^x^See [90]. Japan has two definitions of death; neurological criteria are used for people who have previously consented to organ donation and cardio-respiratory death for everyone else.

^y^The origins of Tibetan Buddhism are unclear. There are features of Hinduism and both Chinese and Indian Buddhism in the tradition found in Tibet.

^z^The actual title is *Bardo Thodol*; it is typically translated as the liberation through hearing during the intermediate states.

^aa^Tsomo’s work has been influential in the wider Buddhist community. Buddhist scholar Damien Keown, once one of the few Buddhist proponents for accepting neurological criteria in determining death, has now changed his position after being asked to review Tsomo’s book on Buddhism and bioethics ([3], p.1). Keown now calls for a robust set of tests that include the cardiovascular, respiratory, and nervous systems to determine death in accordance with Buddhist thought ([3], p.1).

^bb^Not all convert practitioners of these traditions are alike, however. American born Tibetan nun and Buddhist scholar, Karma Lekshe Tsomo exemplifies the convert who holds firmly to the original form of their new tradition. Tsomo’s form of Tibetan Buddhism closely resembles what one might find in Tibet and indeed she studied there.

^cc^Western Zen Buddhism offers many resources relating to death and dying but these do not include discussions about determining death or withdrawing treatment. See the website of the Upaya Zen Center located in Santa Fe, New Mexico [97].

^dd^The controversy about, confusion with, and difficulty with neurologically based definitions of death in Japan illustrates this. Scholars from several fields have discussed the reasons for resistance in Japan; Jīro Nudeshima proffers the theory that the difficulty arises from distrust of the medical profession while philosopher Ominé Akira argues that animism is at the root of the controversy [72]. Margaret Lock and John McConnell both note that acceptance of medical definitions, new imaging technologies, and reproductive technologies have met little resistance in Japan. Lock writes that anxiety about the management of death apparently threatens the social order as most other forms of medical intervention do not [72].
